# Therapeutic extraction of second molars in orthodontics: a scoping review

**DOI:** 10.1186/s12903-024-05346-8

**Published:** 2025-01-06

**Authors:** Ziad M. Montasser, Mona A. Montasser

**Affiliations:** 1 Faculty of Dentistry, Horus University in Egypt, New Damietta City, Egypt; 2 Department of Orthodontics, Faculty of Dentistry, Mansoura University, Mansoura, 35516 Egypt

**Keywords:** Orthodontics, Extraction, Second Molars

## Abstract

**Objective:**

To review the literature to identify the present evidence on the extraction of second molars in orthodontics.

**Materials & method:**

A search of the MEDLINE/PubMed^®^, Scopus, Web of Science™, and ProQuest^®^ databases for full-text articles was done on March 5, 2024. The search went back till the 1st of January 1991 and was limited to articles in English. The results of the first search went through a preliminary check to remove duplicates and then the titles and abstracts of the articles were read to exclude the irrelevant studies, case studies, or reviews. The abstracts of the selected studies were read carefully to verify if the inclusion criteria were met. Finally, the full texts of the potentially eligible studies were read to apply the eligibility criteria and decide whether to include them in the review or not. The eligibility criteria were set following the PICO (population, intervention, comparison, and outcome) standard. The included studies were thoroughly summarized by extracting the most important information.

**Results:**

The electronic search located a total of 103 articles distributed among the databases. Removing duplicates left 48 Studies. After careful assessment of the titles and abstracts, 32 studies were excluded leaving 16 studies. Applying the inclusion/exclusion criteria resulted in the exclusion of 2 studies and including 14 studies in the scoping review.

**Conclusions:**

There is quite low level of evidence to support second molar extraction in orthodontics. Conducting a systematic review would not add much to the evidence as well-conducted RCTs are needed first.

## Introduction

The most common tooth to be extracted as part of an orthodontic treatment plan is the premolar and more specifically the first premolar. Bishara et al. listed three main reasons why the first premolar is the tooth preferred for extraction by orthodontists. These reasons are: (1) excluding the first permanent molar; it is usually the first permanent posterior tooth to erupt, (2) its extraction frees (or hastens) the eruption of the permanent canine, and (3) its position in the center of each quadrant makes the extraction space equally close to the anterior or posterior crowding [1]. The extraction of molars can be an alternative; each molar, first, second, or third, has specific indications for extraction as a component of the orthodontic treatment plan.

Second molar extraction is one of the options during orthodontic treatment. Extraction could be due to reasons related to the molars themselves when the molars are severely carious, have large restorations or root canal filling, or when they are ectopically erupted or severely rotated, but may also be part of the orthodontic treatment plan [2]. Extraction of second molars might be a part of the orthodontic treatment plan specifically in open bite cases and cases that need to create space by first molar distalization [1, 2]. Among the reasons that orthodontists are reluctant to extract molars when therapeutic orthodontic extraction is required is their familiarity with managing premolar extraction contrary to their limited experience with managing molar extractions [3].

Chua and Felicita [4] reviewed the literature and summarized the indications for extraction of the second molars. They found that extraction of maxillary second molars could be used for the treatment of Class II cases and cases with moderate maxillary arch crowding. Mandibular second molars are indicated for extraction in Class III cases. Extraction of maxillary second molars in Class II cases and mandibular second molars in Class III cases showed favorable soft tissue changes. Extraction of the four second molars is indicated to facilitate distalization of the first permanent molar and in cases with anterior open bite as it reduces the functioning molar area and moves the mandibular fulcrum one molar forward.

Third molars could be used as a substitute for the extracted second molars; in such a case it required that third molars be in a good position to indicate their future eruption in good alignment and proper occlusion [5]. As early as 1977, Liddle tried to draw attention to the effects of the erupting second and third molars that may cause malocclusion. Liddle tried to use the extraction of all four permanent second molars as an interception measure to avoid the forward force of eruption as he considered extracting the premolars in many cases could be a treatment of the effect rather than removing the cause [6].

Assessing the records of 3,413 patients treated from 1973 to 2007 in one university orthodontic clinic by creating 10 protocols according to the pattern of extraction and dividing the 35 years into 7 intervals of 5 years each found that generally, frequency of non-extraction treatment increased on the expense of the extraction frequency. One of the 10 protocols assessed in the study was “first or second molar extractions” and it showed low frequency (about or less than 1.0%) in the 5 intervals started in 1983 suggesting no significant changes in the indications of their extraction. Before 1983 there were no records of cases treated by the “first or second molar extractions” [7].

Over the years, many case studies have been published about the extraction of the second molars in orthodontic treatment. Case studies presented extraction of second molars alone or in addition to the commonly used protocol of premolars extraction. The case studies included extraction of second molars in the treatment of bimaxillary protrusion [8], Class II [9, 10], Class III malocclusion [11, 12], and space gaining and relief of crowding [13, 14]. The decision for extraction takes into consideration the malocclusion, the objectives of treatment, and the mechanics to be used [15].

To the best of our knowledge, this is the first scoping review that specifically investigated the literature on the extraction of second molars in orthodontics. This scoping review was carried out to fill, or help fill, a knowledge gap about the evidence on the extraction of second molars in orthodontics.

## Materials and methods

### Databases search and study selection

**Fig. 1:**
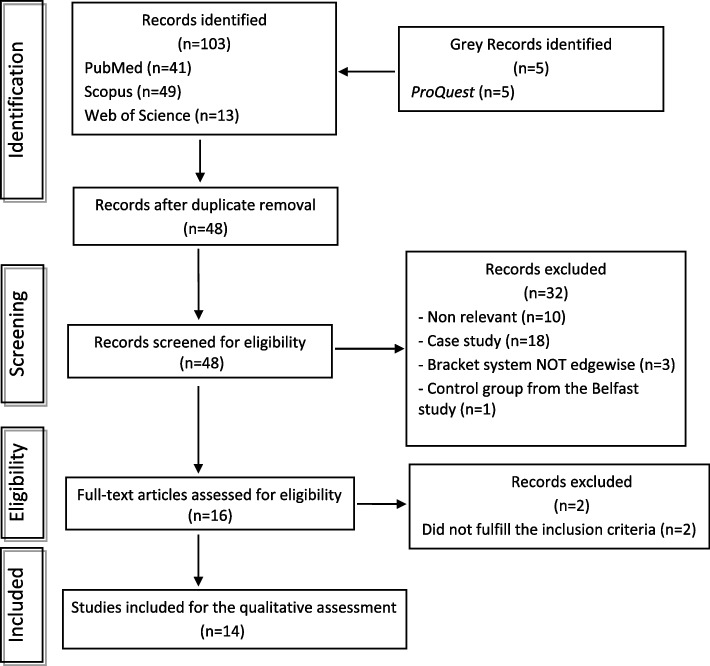
Flow diagram for screening and selection of the sources

To conduct this scoping review, a search of the databases of the electronic literature for full-text articles was done on March 5, 2024. The search went back about three decades specifically till the 1st of January 1991. The databases searched were MEDLINE/PubMed^®^, Scopus, Web of Science™, and ProQuest^®^. The search was limited to articles in English. The Boolean operators “AND” and “OR” were used to form combinations of the keywords. The terms used in the search and modified for the different databases were «orthodontic*» OR «malocclusion*» AND «second molar extraction*». The results of the first search went through a preliminary check by an author to remove duplicates using Excel^®^ sheet then, the titles and abstracts of the articles were read to exclude the irrelevant studies, case studies, or reviews. Then the abstracts of the studies selected in the first stage were read carefully to verify if the inclusion criteria were met. Finally, the full texts of the potentially eligible studies were read to apply the eligibility criteria and decide whether to include them in the review or not (Fig. 1).

## Eligibility criteria

Following the PICO (population, intervention, comparison, and outcome) standard, the following eligibility criteria were applied:

1. **Study design**: Prospective or retrospective controlled, comparative studies, or single-arm studies.
2. **Participants**: Orthodontic patients who have their second molars extracted.
3. **Interventions**: Extraction of second molars with or without fixed edgewise appliance.
4. **Comparator**: Comparison group with a different extraction pattern or with different/no intervention. Single-arm studies were also considered.
5. **Outcomes**: Treatment changes, spontaneous changes, third molar eruption, alignment and occlusion, treatment duration, and stability.

### Data collection

The full texts of the studies that met the inclusion criteria were thoroughly read carefully and the most important information that summarizes the paper materials and methods, results, and conclusions was extracted and organized in tables. The information extracted included; the study’s first author’s name and year of publication, study design, participants, study groups, intervention(s), outcome(s), and outcome measure(s) (Table 1). Throughout the work, the two authors worked independently and disagreements were resolved by discussion.

**Table 1:** Summary of characteristics of studies included in the review

	Study	Study design	Intervention	Comparison	Outcomes	Assessment	Treatment/Follow-up
1	Færøvig et al., [16] 2024	Retrospective Comparative Study	Extraction of second molars + Fixed appliance	Cases: Class II predominantly and Class I: Group 1: Extraction of mandibular second molars with fixed appliance (*n* = 68) Group 2: Control [no mandibular fixed appliance (*n* = 86)]	- Occlusal changes	- Peer Assessment Rating (PAR index) - American board of orthodontics (ABO) grading system	- Follow-up: Till eruption of mandibular third molar (mean = 6 ± 2 years).
2	Paddenberg et al., [17] 2023	Retrospective Comparative Study	Extraction of second molars + Fixed appliance	Cases: Class II: Bilateral extraction of: Group 1: Maxillary second molars (*n* = 31) Group 2: First premolars (*n* = 22)	- Treatment timing, - Cephalometry, - Maxillary third molar alignment - Relapse in the long-term	- Time in months. - Angular and linear cephalometric measurements	- Treatment time: Group 1: 24 ± 5.9months Group 2: 21.9 ± 3.6months Retention: 6-7years post treatment.
3	Kim et al., [18] 2023	Retrospective	Extraction of second molars	One group (*n* = 87 patients, 136 maxillary third molars)	- Occlusal status of maxillary third molars:	- Study models Alignment, marginal ridge discrepancy, occlusal contact, interproximal contact, and buccal overjet.	- At the time of full eruption of third molars (T1).
					- Factors influenced the third molars eruption:	- Panoramic & Cephalometric radiograph - Third molars: Nolla’s stage, long axis angle, vertical and horizontal position of the maxillary third molar, and the maxillary tuberosity space.	- At the time of extraction of second molars (T0) and at full eruption of third molars (T1).
4	Kato et al., [19] 2022	Retrospective	Extraction of second molars	One group (*n* = 84 maxillary third molars)	- Late maxillary third molar eruption	- Panoramic radiograph - Cephalometric radiographs	- Before extraction of second molars (T0) and at full eruption of third molars (T1). > 500 days after Maxillary second molars extraction
5	Kojima et al., [20] 2009	Prospective Comparative Study	Extraction of second molars + Fixed appliance	Cases: Open bite previously treated without extraction: Group 1: Maxillary second molars extraction (*n* = 15) Group 2: Non-Extraction (*n* = 15).	- Dentoskeletal morphological changes	- Angular and linear cephalometric measurements	- Treatment Time: Group 1: 2.0 ± 0.8 years. Group 2: 1.7 ± 1.0 years.
6	De-la-Rosa-Gay et al., [21] 2006	Retrospective	Extraction of second molars	One group (*n* = 48 patients) or (*n* = 128 third molars; 54 maxillary and 74 mandibular)	- Eruption of third molars	Panoramic radiograph - Third molars: -Nolla developmental stage at (T0) The angle between the third molar and the neighboring first molar at (T0) and (T1)	- Before extraction of second molars (T0) and at full eruption of third molars (T1). Median 3-4Years
					- Factors associated with unsuccessful third molar eruption	- Third molars: Nolla developmental stage at (T0). The angle between the third molar and the neighboring first molar at (T0) and after the third molar was considered “impacted” with fully formed roots.	
7	De-la-Rosa-Gay et al., [22] 2010				Predictive models: - Lineal regression model for the angle between the third and the adjacent first molar at (T1) - Logistic regression model for the successful eruption for the third molar.	The independent variables: - Site of the molar (maxillary. or mandibular), sex, age, and Nolla’s stage at (T0), the angle between the third and the corresponding first molar at (T0) and first-degree interactions.	
8	Waters and Harris [23], 2001	Retrospective Comparative Study	Extraction of second molars + Fixed appliance	Cases: Class II: Group 1: Maxillary second molars extraction (*n* = 25) Group 2: Non-Extraction (*n* = 25).	- Dentoskeletal morphological changes	- Angular and linear cephalometric measurements	- Treatment time: Group 1: 2.0 ± 0.9years Group 2: 2.7 ± 0.0years
9	Moffitt [24], 1998	Retrospective	Extraction of second molars + Fixed appliance	Cases: Not specified 56 patients treated with extraction of maxillary second molars - Unilateral extraction (*n* = 28) - Bilateral extraction (*n* = 28 ) - Unilateral extraction (*n* = 28) Served as split mouth randomized sample to assess time of eruption.	- Eruption and function of third molars	- Age of eruption. Clinical, Bitewing, Cepalometric, and Study model changes of third molar position & interproximal periodontal health	- Follow-up: 2–101 months post-treatment.
10	Battagel and Rayan [25], 1998	Retrospective	Extraction of second molars + - “en masse” appliance and extraoral traction, followed by edgewise mechanics in the maxillary arch - No intervention in the mandibular arch (Spontaneous eruption)	Cases: Class I crowding or mild Class II Group 1: Mandibular second molars extraction (*n* = 18) Group 2: Non-Extraction (*n* = 23).	Spontaneous changes in the mandibular arch including: - Intercanine and intermolar widths. - Arch length and perimeter. - Crowding in the labial segment and whole arch.	- Study models.	- Start of treatment, - Completion of buccal segment retraction, - Completion of active appliance therapy.
11	Basdra et al., [26] 1996	Retrospective Single arm Study	- Extraction of second molars	Cases: Class II One group (*n* = 32): Extraction of maxillary second molars	- Dentoskeletal morphological changes	- Cephalogram (18 linear&angular)	- After treatment
				Subgroup of (*n* = 19 patiets, 34 maxillary third molars)	Third molar changes axial inclination	- Panoramic radiograph	− 4 years post retention
					Third molar - Form - Position (occlusion&rotation)	- Study models	
					Third molar - Periodontal status (Pocket&mobility)	- Clinical Evaluation	
12	Richardson [27], 1996	Retrospective Single arm Study	- Extraction of second molars + Fixed appliance in the maxillary arch - No intervention in the mandibular arch	Cases: Class I crowding or mild Class II One group (*n* = 30): Extraction of four second molars at average age of 13.9 years - None had treatment in the mandibular arch − 20 had treatment in the maxillary arch	- Long term mandibular arch alignment	- Study models.	- After 5 and 10 years of second molars extractions
13	Richardson and Richardson [28], 1993	Prospective Single arm Study	- Extraction of second molars	Cases: Not Specified One group (*n* = 63): Extraction of mandibular second molars	- Stage of development and M-D&B-L angulation of third molars	- 60˚ cephalograms	- Before extraction and 3–10 years later.
					- Subjective evaluation of the final third molars position	- Study models.	- After 3–10 years of treatment.
14	Richardson and Burden [29], 1992	Retrospective	- Extraction of second molars + Removable or fixed appliance	Cases: Crowding in the premolar area Group 1: Mandibular second molars extraction (*n* = 13) + Fixed appliance. Group 2: Mandibular second molars extraction with no appliance treatment (*n* = 21). Both groups had appliance in the maxillary arch	- Relief of the crowding in the premolar area	- 60˚ cephalograms - Panoramic radiograph - Study models	- Before extraction and at end of treatment.

## Results

### Study selection

The electronic search located a total of 103 articles distributed among the databases; PubMed^®^ (*n* = 41), Scopus (*n* = 49), Web of Science™ (*n* = 13), and ProQuest^®^ (*n* = 5). Removing duplicates left 48 Studies. After careful assessment of the articles from the titles and abstracts to assess the relevance of the studies to the review topic, 32 studies were excluded leaving 16 studies with potential eligibility to be included in the review. The reasons for excluding the studies were; non-relevant (*n* = 10), case study (*n* = 18), bracket system not edgewise (*n* = 3), and control group from the Belfast study (*n* = 1). The full texts of the selected 16 studies were carefully read to apply the inclusion/exclusion criteria which resulted in excluding 2 studies and including 14 studies [16–29] in the scoping review. The flowchart (Fig. 1) of the study summarizes the steps and the results.

### Study characteristics

Out of the 14 included studies, only 2 studies were prospective [5, 13] and 7 studies [2, 4, 6, 7, 11–13] included only one group. Studies on the extraction of the mandibular second molars were less than those on the extraction of the maxillary second molars. Although, the extraction of the mandibular second molars was investigated in 7 studies [16, 21, 22, 25, 27–29], the two studies by De-la-Rosa-Gay et al. [21, 22] as well as the three studies by Richardson [27], Richardson and Richardson [28], and Richardson and Burden [29] were based on the same sample.

## Discussion

Extractions for othodontic treatment continue as it is considered by some as a “standard of care”, Weintraub et al. [15] found a significant difference between the rates of extraction reported by clinics subjectively and those based on an objective review of the records from the same clinics. The subjective rates ranged from 5 to 87% while the actual rates ranged from 25 to 85%.

Chua and Felicita [4] reviewed the literature and summarized the indications for extraction of the second molars; extraction of maxillary second molars could be used for the treatment of Class II cases and cases with moderate maxillary arch crowding while mandibular second molars are indicated for extraction in Class III cases. Extraction of maxillary second molars in Class II cases and mandibular second molars in Class III cases showed favorable soft tissue changes. Extraction of the four second molars is indicated to facilitate distalization of the first permanent molars and in cases with an anterior open bite to reduce the functioning molar area and move the mandibular fulcrum one molar forward.

The search for the current systematic review showed a large number of publications that presented a vast variability for using extraction of second molars in the treatment of orthodontic cases However; a large portion was case studies. Finally included studies in the current scoping review focused on third molar eruption and alignment [17, 19, 21, 22, 24, 26, 28], and occlusal changes in the dental arches [16–18, 26] after second molars extraction, dentoskeletal morphological changes [20, 23, 26], stability of lower arch alignment [27], relief of posterior crowding [29], and duration of treatment [17].

### Eruption, alignment, and occlusion of the third molars

The second permanent molars are important for proper occlusion, correct occlusal vertical dimension, and the relationship between the condyle and the glenoid fossa [30]. Therefore, a major concern when considering the extraction of the second molars for orthodontic purposes was the eruption of the third molars and their final alignment, occlusion, and function. Based on that, one of the most frequently evaluated outcomes after extraction of the second molars for orthodontic reasons was the eruption of the third molars. Third molars could be used as a substitute to extract second molars; in such a case it is required that third molars be in a good position indicating their future eruption in good alignment and proper occlusion. De-la-Rosa-Gay et al. [21] defined the successful eruption of a third molar by its eruption in proximal contact with its adjacent first molar with an angle between them of ≤ 35° while, Kim et al. [18] considered the third molar successfully erupted when it is in contact with its antagonist or when the marginal ridge discrepancy with the adjacent first molar was within 0.5 mm.

Kato et al. [19] found that eruption of the maxillary third molars with complete or almost complete root formation occurred in about 96% of the cases. According to Moffit [24], in about 92%, 97%, and 69% of the cases the maxillary third molar will erupt with acceptable mesiodistal angulation, buccolingual angulation, and rotations respectively. Basdra et al. [26] reported 100% eruption of the followed-up maxillary third molars; all the followed-up maxillary third molars erupted into occlusion with a mesial contact point with the adjacent first molar and acceptable mesiodistal axial inclination over 4 years of follow-up. They stressed the importance of evaluating the size and form of the third molar because a malformed third molar is not a suitable substitute for the extracted second molar. Extraction of the second molar generally accelerates the eruption of the adjacent third molar [19, 24]; delayed eruption (≥ 500 days after extraction of the maxillary second molars) was associated with the proximity of the maxillary third molars roots to the sinus floor, the distance between their occlusal plane and the apical third of the maxillary second molar roots, the ANB angle, and the distal movement of the maxillary first molar after extraction of the adjacent second molar. De-la-Rosa-Gay et al. [21] reported 66.2% successful eruption of the mandibular third molars after extraction of the adjacent second molars before or during orthodontic treatment; most of the unsuccessful eruptions were due to high mesial tilting or lack of proximal contact.

The higher the Nolla’s developmental stage of the maxillary or mandibular third molar at the time of extraction of the adjacent second molar potentially delays its eruption [19, 21].

### Dentoskeletal morphological changes

Second molars have been extracted over the years for the treatment of skeletal malocclusions. Extracting the second molars results in significantly different dentofacial changes compared to the traditional extraction of first premolars; Paddenberg et al. [17] found less significant profile changes including dental, skeletal, and soft tissue changes when maxillary second molars were extracted compared to first premolars extraction. Basdra et al. [26] Extracted the maxillary second molars for treatment of 32 Class II patients who showed significant dental, skeletal, and soft tissue changes favorable for the Class II treatment after the extraction; the ANB angle decreased, the maxillary incisors retroclined, and the upper lip protrusion improved while the posterior to the anterior facial heights decreased.

Greatrex et al. [31] in a retrospective study examined the effect of the extraction of four second molars on the dentoskeletal changes in borderline cases that bare little facial changes. Most of the cases were Class I and treatment mechanics was Tip-Edge appliance. The lower incisors revealed a tendency to remain in their pre-treatment sagittal positions. The study did not include a control group so further studies were recommended. Another study used the Tip-Edge mechanics and evaluated the dentofacial profile changes after extraction of lower second molars in the treatment of severe Class III subjects who refused orthognathic surgery. Changes in measurements from lateral cephalometric radiographs suggested that success in the treatment of some severe Class III permanent dentition can be achieved with fixed appliances and extraction of lower second molars. Favorable soft-tissue changes were noted, and the concave facial profile changed to a straight profile [32].

Iijima et al. [8] published a case study that presented a clinical application of second molar extraction for the treatment of Class II in an adult patient. Second molars were extracted in addition to the extraction of the first premolars in the orthodontic treatment of a bimaxillary protrusion case. Extraction of maxillary second molars allowed first molars distalization. Although third molars were of not of perfect form or position, they erupted and the dentition was stable after two years of retention. Extraction of second molars to treat Class II is a tricky decision; Isaacson et al. [33] presented a case that was treated by a general practitioner where extraction of all four second molars was an incorrect decision. In the presented case, it was impossible to distalize the first molars to correct the 75% full Class II and resolve 10 mm maxillary and 6 mm mandibular crowding. After re-treating the case with extraction of maxillary lateral incisors; follow-up showed potential for successful eruption of the third molars to replace the extracted second molars.

### Treatment of mandibular crowding

Although dental crowding may be independent of the skeletal measurements [34], the uniqueness of the mandible influences planning orthodontic treatment; extraction sometimes is the best option to relieve mandibular crowding considering the limited ability to change the dimensions of the mandible [35].

Faerøvig et al. [16] evaluated long-term spontaneous occlusal changes in the lower arch with mild mandibular crowding following extraction of second molars by comparing the study models of two groups, both have maxillary fixed appliance while a subgroup had fixed appliance and the second acted as control. Evaluation over 6 years follow-up starting before extraction of lower second molars (9–16 years old) and continuing till eruption of lower second molars (14–25 years old) showed similar changes of mild mandibular crowding. Favorable occlusal outcomes of the lower third molars were observed in about 82% of cases with no difference between the two groups. In another study by Battagel and Ryan [25], the spontaneous changes that occurred in the lower arches were compared between two groups that had orthodontic appliance in the maxillary arch while; one group had the mandibular second molars extracted while they were maintained in the second group. Models were examined at the start of treatment, after buccal segment retraction, and when active appliance therapy was complete. Over 2-years of follow-up; arch length, arch perimeter, and crowding showed significantly different changes between the two groups. Spontaneous changes were seen in the lower arch, despite the absence of any therapy, crowding was slightly improved and inter-molar width increased, apparently as a response to the expansion in the maxillary arch. At the end of treatment, upper arch retraction and expansion were reduced as the teeth were integrated with the lower dentition: the canines required less than 1 mm further retraction. In the lower arch, the expansion of the molars was essentially stable.

Richardson and Burden [29] included thirty-four young patients with mandibular premolar crowding treated by extraction of second molars in a study where 13 patients had mandibular arch orthodontic treatment started not less than 6 months after the extractions. The rest of the group, 21 patients, acted as a control with no treatment in the mandibular arch. The study found that extraction of the mandibular second molars with or without orthodontic appliance can lead to relief of 4–5 mm of mandibular premolar crowding and although, it may not be possible to predict accurately which patients will require active appliance therapy, early extraction of lower second molars before second premolar eruption is suggested to be favorable for spontaneous premolar alignment.

### Late mandibular crowding

Extracting the lower second molars, to intercept mandibular crowding, without using any orthodontic appliance in 30 adolescent patients led to a small average decrease in lower arch crowding measured on study models in the first five years following extraction, and there was little or no change in the alignment in the following five years of follow-up [27].

### Treatment duration

The treatment duration did not differ significantly between the group that had their first premolars extracted and the group that had the second molars extracted [17].

## Limitations

Systematic reviews on orthodontic topics usually face difficulty in securing a decent number of RCCT or CCT studies for inclusion. The number of RCTs or CCTs found for this scoping review was very small. Another limitation was the quality of the studies found eligible for inclusion. The quality was compromised by multiple factors related to study design, randomization, and data reporting. Sources of bias related to the design of the studies could be attributed in large to being mostly retrospective studies. Retrospective studies usually do not include a homogenous population regarding the age, gender, or number of subjects. Also, in retrospective studies the intervention is usually assumed, intuitively, to be chosen by the patient or jointly by the patient and the orthodontist and therefore cannot be considered randomly distributed, and vast variations in the outcome assessment are usually noted. Data reporting is also a concern as data was missing in many studies about factors such as gender distribution, the type of malocclusion, and the mechanics used.

## Conclusions

1. Studies on the extraction of second molars in orthodontics looked mostly at the effect on the: third molars, occlusal changes, dentoskeletal morphological changes, stability of lower arch alignment, relief of posterior crowding, and duration of treatment.
2. The number of studies looked at any of the aspects mentioned above is small and shows high methodology variability.
3. Based on the results of this scoping review there is quite low evidence to support the reliability of second molar extraction in orthodontics.
4. Conducting a systematic review would not add much to the evidence as well-conducted RCTs are needed first.

## Data Availability

No datasets were generated or analysed during the current study.
